# Low-Cost IoT-Based Predictive Maintenance Using Vibration

**DOI:** 10.3390/s25216610

**Published:** 2025-10-27

**Authors:** Peter Kolok, Michal Hodoň, Peter Ševčík, Léo Hotz, Nicolas Remy

**Affiliations:** 1 Department of Technical Cybernetics, Faculty of Management Science and Informatics, University of Žilina, Univerzitna 8215/1, 010 26 Žilina, Slovakia; michal.hodon@fri.uniza.sk (M.H.); peter.sevcik@fri.uniza.sk (P.Š.); 2 Le Cnam En Grand Est, 4 Avenue du Docteur Heydenreich-BP 65228, 54052 Nancy, France

**Keywords:** predictive maintenance, MEMS sensors, ESP32, vibration analysis, acoustic monitoring, anomaly detection

## Abstract

Predictive maintenance helps reduce operational costs and improve machine reliability by anticipating failures. However, existing solutions are often too expensive or complex for small rotating machinery such as fans or low-power motors. This work presents a low-cost, IoT-based monitoring system using an ESP32 microcontroller combined with MEMS sensors (an accelerometer and a microphone). The system continuously collects vibration and acoustic signals, which are then processed using RMS and FFT techniques. Machine learning algorithms, such as anomaly detection or basic classification, are used to identify deviations from normal operation. A working prototype was tested under various fault conditions, including imbalance and wear. The system successfully identified abnormal states through signal deviations in both time and frequency domains, with over ~73% detection accuracy. The proposed solution is cost-effective, simple to implement, and well-suited for educational or industrial environments. It demonstrates the potential of embedded systems and basic signal analysis for scalable predictive maintenance applications.

## 1. Introduction

PdM has emerged as a pivotal strategy in the Industry 4.0 era to reduce unplanned downtime and increase equipment availability. By leveraging connected sensors and data processing at the edge or in the cloud, PdM enables early detection of machine degradation. Recent MDPI reviews emphasize the central role of vibration-based sensing using accelerometers, as well as the growing importance of acoustic signals for identifying early fault patterns. The widespread adoption of MEMS sensors—characterized by their low cost, low power consumption, and ease of integration—makes these techniques accessible even beyond heavy industrial contexts [1,2,3].

For small actuators such as fans and micro-motors, existing commercial PdM solutions are often oversized and prohibitively expensive. In contrast, low-cost IoT architectures based on ESP32 nodes equipped with MEMS sensors, typically a triaxial accelerometer combined with a microphone, can provide continuous monitoring. These systems enable on-device extraction of time- and frequency-domain features such as RMS, FFT, and spectra, while transmitting only selective alerts. Recent studies have demonstrated the feasibility of embedded signal processing on ESP32-class microcontrollers for vibration and acoustic monitoring, allowing local decision-making complemented by cloud analysis where required [4,5,6].

Sensor calibration has been another focus of research. Studies have confirmed that MEMS accelerometers, when properly calibrated and operated within suitable bandwidths, achieve adequate sensitivity for bearing diagnostics in PdM applications. MEMS microphones provide complementary information by capturing phenomena that are less visible in vibration signals, including friction, air leakage, and incipient faults. These signals can be analyzed effectively through spectral representations such as the STFT and spectrograms, while imposing only modest computational overhead [2,7,8].

Fault detection in PdM often relies on lightweight unsupervised learning techniques. Among these, IF has gained attention for its robustness, linear-time complexity, and minimal hyperparameter requirements. It is particularly well-suited for distinguishing anomalous signals from healthy baselines. Although variants such as Extended Isolation Forest exist, the standard IF remains a strong compromise for embedded deployment, including on ESP32 devices [9].

Based on these insights, the present work proposes an edge-computing pipeline for PdM that integrates MEMS data acquisition, preprocessing (RMS, FFT, and filtering), compact feature vectorization, Isolation Forest anomaly detection, and selective reporting of anomalous segments to a server for traceability. The objective is to demonstrate that PdM can be affordable, reproducible, and effective on small rotating systems by employing a low-cost ESP32 + MEMS node capable of vibration and acoustic sensing, feature extraction, and on-device anomaly detection [4,6].

The remainder of this paper is organized as follows. Section 2 reviews related work on vibration- and acoustic-based predictive maintenance systems and outlines the state of the art in MEMS sensing and edge computing. Section 3 describes the materials and methods, including the hardware setup, ESP32 firmware architecture, and Isolation Forest anomaly detection pipeline. Section 4 presents the experimental results and evaluates the diagnostic performance of the proposed system. Finally, Section 5 concludes the paper and discusses future research directions toward fully embedded, multimodal predictive maintenance frameworks.

## 2. Related Works

PdM and smart sensing technologies have been the subject of extensive research in recent years. MDPI review papers emphasize the maturity of smart sensor systems and data-driven diagnostic pipelines, covering sensor types, edge/cloud architectures, and data workflows. They converge on the effectiveness of a low-cost local node for early anomaly detection, provided that features and thresholds are carefully selected [1]. Recent surveys further highlight the importance of intelligent sensors and IoT-based monitoring frameworks for predictive maintenance in smart factory environments [2,3,4,5,6,7,8,9,10,11,12,13,14,15,16,17]. Complementary system-level views also stress PdM as a preventive layer in IoT-monitored infrastructures, where data pipelines and maintenance scheduling are co-designed to reduce fault rates [18]. Recent studies additionally explore hybrid edge-cloud orchestration for fleet-level monitoring and failure detection, showing how inference can be partitioned between local devices and cloud services to improve latency and scalability [19,20].

The authors in [21,22,23] focused on the use of MEMS accelerometers for vibration analysis, showing that although these devices are noisier than piezoelectric sensors, they provide measurement accuracy compatible with PdM within useful frequency bands (up to several tens of kHz depending on the model). Their studies highlighted the importance of dynamic calibration, which improves the fidelity of diagnostic metrics such as RMS, harmonic peaks, and signal envelopes. These works further confirmed the sensitivity of MEMS accelerometers to bearing and friction faults. Recent reviews sharpen this perspective by mapping vibration techniques to concrete rotating-machinery scenarios and reporting practical ranges for low-cost sensing stacks [1]. Noise-aware pipelines and feature selection remain critical when deploying low-cost sensors in industrial environments, especially where operating conditions vary [24,25].

In [26], the authors demonstrated that MEMS microphones are increasingly adopted in PdM because of their low cost and ease of implementation. They showed that these microphones can capture subtle anomalies using spectral or time-frequency analysis. Their review further emphasized that vibro-acoustic fusion improves detection performance and robustness, particularly in small machinery contexts. More recent studies confirmed that MEMS microphones combined with lightweight neural models can reliably diagnose induction motor faults [27], while other reviews underline the growing role of sound-based sensing as a complementary modality to vibration analysis [24]. Further, recent works survey audio/IoT sensing in modern manufacturing and emphasize robustness to environmental noise and sustainability-driven deployments [25,28,29].

Research in [2,19] described the role of signal processing methods as a foundation for PdM. Techniques such as FFT, power spectral density, band-pass filtering, RMS, and higher-order statistics remain central to extracting fault signatures, including imbalance, misalignment, or bearing defects. These methods were shown to be embeddable on microcontrollers, while image-like representations such as spectrograms can also be explored with visual classifiers, even though they are not essential in proof-of-concept implementations with limited computational resources. Recent reviews of belt-conveyor idler monitoring consolidate vibration- and acoustic-feature practices (e.g., SK, MFCC, and time-frequency mappings) and compare ML models across datasets and operating regimes [24].

The authors in [3,30] investigated anomaly detection and highlighted the effectiveness of lightweight machine learning techniques. Isolation Forest was identified as particularly suitable due to its simplicity and scalability in unsupervised outlier detection, operating directly on healthy data without the need for labeled fault data. While extensions such as Extended IF exist, the basic Isolation Forest provides the best trade-off between accuracy and computational efficiency in embedded environments. In parallel, distributed and on-device neural approaches have been proposed to scale anomaly detection across edge devices without centralizing raw data, supporting privacy and bandwidth constraints [31].

In [4,32,33], several authors explored edge computing approaches with ESP32 nodes, which were employed for multi-sensor data acquisition (vibration, temperature, and acoustics), local preprocessing (e.g., FFT), and either local or cloud-based decision-making. Their findings confirmed the feasibility of combining ESP32 and MEMS devices within an embedded processing and network-integrated architecture for PdM. Recent demonstrations extend this line to process-control contexts (e.g., biogas reactors) using ESP32-based low-cost nodes, confirming practical viability outside laboratory settings [34]. Beyond single-asset setups, hybrid edge-cloud frameworks for real-time PdM illustrate partitioned inference and centralized model management for industrial equipment [19,20,35]. In parallel, digital-twin workflows show how virtual models of AC machines can be coupled with sensor data to improve diagnostics and maintenance planning [36]. Generalizable ESP32-class edge patterns (e.g., compressed models and task-specific pre-processing) further demonstrate how object-detection/vision pipelines can be adapted to time-series PdM on constrained hardware [37], and PLC-integrated PdM confirms feasibility in legacy industrial control systems [38].

A recent MDPI survey [5] reviewed hand-crafted features for condition monitoring and listed 169 documented time- and frequency-domain metrics. These features were shown to be interpretable, computationally efficient, and well suited for edge-based fault diagnosis on resource-constrained hardware. More recent works continue to highlight the development of low-cost embedded PdM systems. For instance, ref. [6] presented autoencoders for vibration-based anomaly detection, ref. [7] applied machine learning to induction motor vibrations, ref. [8] implemented lightweight deep learning for vibration diagnosis at the edge, and ref. [9] demonstrated remote vibration monitoring using ESP32-class devices. Similarly, ref. [10] investigated acoustic-based monitoring for CNC machines, confirming the growing role of MEMS microphones in PdM. Altogether, these contributions reinforce that PdM research is shifting strongly toward embedded, low-cost solutions that combine vibration and acoustic sensing with edge intelligence [24,27,35,39]. This trend is mirrored by broader edge/fog deployments and sustainability-aligned industrial audio sensing [20,28].

Several open datasets have been widely adopted for reproducibility and benchmarking. The CWRU Bearing Data Center remains the most used dataset for vibration-based fault detection. The IMS dataset (NASA/University of Cincinnati) provides long run-to-failure experiments, while the PU dataset (Paderborn University) contains controlled bearing faults under different loads and speeds. More recent contributions include the XJTU-SY dataset of accelerated bearing life tests and the PRONOSTIA/IEEE PHM 2012 dataset, which has become central to prognostics challenges [5]. These datasets now serve as reference points for anomaly detection and remaining useful life estimation methods. Recent acoustic anomaly detection studies also report noise-robust multiclass setups evaluated on public/industrial recordings, underscoring the need for standardized audio benchmarks [25].

From this analysis, it is evident that the literature strongly supports a low-cost PdM strategy built on several complementary elements. First, multiple studies have shown that calibrated MEMS accelerometers and microphones provide sufficient sensitivity for vibration and acoustic monitoring in rotating machinery, while still maintaining very low hardware costs [22,23,26]. Second, the combination of statistical feature extraction with lightweight anomaly detection algorithms—most notably Isolation Forest—has been repeatedly validated as a practical compromise between accuracy and computational efficiency [5,32]. Finally, recent works emphasize the importance of edge-computing integration, where devices such as ESP32 nodes perform local preprocessing, selective cloud upload, and on-device decision-making [24,27,35,39], with deployments reported in both industrial equipment and process-control environments [18,34]. Emerging hybrid edge-cloud and decentralized learning schemes further improve scalability and resilience in multi-asset settings [19,20,31]. Taken together, these findings outline a coherent research direction toward affordable PdM systems that combine vibration and acoustic sensing, efficient signal processing, and embedded machine learning. This trajectory aligns precisely with the architecture of our prototype for small machinery.

While multiple studies have explored ESP32- and MEMS-based predictive maintenance systems [5,14,16,30], their approaches differ significantly in mechanical configurations, sensor types, and data collection volumes, which makes direct numerical comparison difficult. The present work, therefore, focuses on the trade-off between resource constraints and diagnostic performance rather than reproducing identical setups. The proposed prototype achieves reliable fault detection at a total cost below 30 EUR, consumes less than 300 mW, and provides anomaly detection results with an average latency below two seconds. These metrics position the system among the most energy- and cost-efficient PdM frameworks for small-scale machinery, especially in decentralized or educational environments where high-performance hardware is not feasible [10,27,39].

Concerning algorithm selection, advanced deep learning methods such as CNN, DQN, and FCIHMRT have demonstrated superior diagnostic accuracy in complex industrial systems [2,8,27,31,37,39]. However, these architectures are computationally demanding, as they require multidimensional inputs (e.g., spectrograms), large convolution kernels, and floating-point inference, which exceed the 512 kB RAM capacity of the ESP32-C6 and significantly increase power consumption [8,37,39]. In particular, DQN and FCIHMRT frameworks involve iterative training and parameter optimization that are incompatible with deterministic, resource-limited edge environments [2,31]. In contrast, the Isolation Forest algorithm adopted in this study provides unsupervised learning capability, linear inference time, and a minimal memory footprint (<150 kB), making it an optimal compromise between accuracy, interpretability, and real-time feasibility for embedded predictive maintenance applications [3,27,30,31,39].

## 3. Materials and Methods

This section describes the experimental setup and firmware architecture of the ESP32-based prototype developed for data acquisition and processing. All hardware components were sourced from commercially available modules, including the following main devices: ESP32-C6 Development Kit (Espressif Systems, Shanghai, China), MPU6050 accelerometer (InvenSense, San Jose, CA, USA), HC-SR04 ultrasonic sensor (Elecfreaks, Shenzhen, China), ADPS9960 proximity sensor (Avago Technologies, San Jose, CA, USA), INMP441 MEMS microphone (InvenSense, San Jose, CA, USA), OLED display (Waveshare Electronics, Shenzhen, China), and L298N H-bridge motor driver (STMicroelectronics, Geneva, Switzerland). Data processing and visualization were performed in Python (Version 3.11.9, Python Software Foundation, USA) using scikit-learn (Version 1.5.2) and Matplotlib (Version 3.9.2) for result visualization.

The following subsections describe the hardware design, including sensor placement, and the firmware modules responsible for motor control, sensing, and data transmission.

### 3.1. Hardware Setup

In this project, a teaching aid in the form of a mobile platform with two DC motors controlled by an H-bridge was used. In addition to basic components such as an LED display, buttons, LEDs, and photoresistors, the key sensing element for this task was the MPU6050, managed by the ESP32-C6 Development Kit, which ensures communication, data processing, and transmission for further analysis. The ESP32 reads motion data from the MPU6050 accelerometer, which is rigidly fixed between the two DC motors of the test bench. The accelerometer was positioned symmetrically between the motors to capture vibrations and instabilities from both sides, and the data was streamed to a laptop over Wi-Fi/TCP at a rate of one line every 0.5 s (≈2 Hz effective sampling). This reduced streaming rate was chosen for simplicity and to limit bandwidth during prototyping. The MPU6050 thus provides valuable information about acceleration, rotation, and orientation, which allows the analysis of dynamic behavior, vibration detection, and the identification of potential faults. By focusing on vibration-based sensing, the ESP32-C6 platform enables a comprehensive evaluation of the system’s condition and supports effective fault detection in the motors.

**Figure 1:**
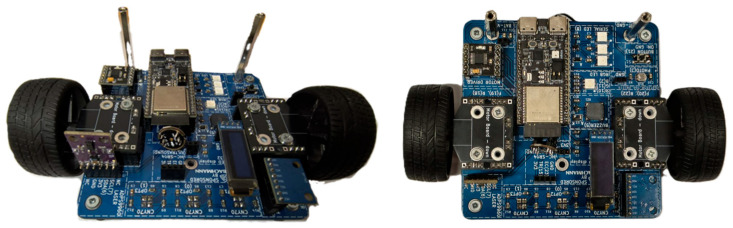
Small Robot Kit with ESP32-C6 DK.

Figure 1 shows the small robotic test platform equipped with the ESP32-C6 Development Kit and MEMS accelerometer MPU6050. The platform serves as a vibration test bench with two DC motors controlled by an H-bridge. The accelerometer is rigidly fixed between the motors to capture mechanical vibrations during operation. This setup allows the acquisition of motion and vibration data for fault detection and dynamic behavior analysis.

**Figure 2:**
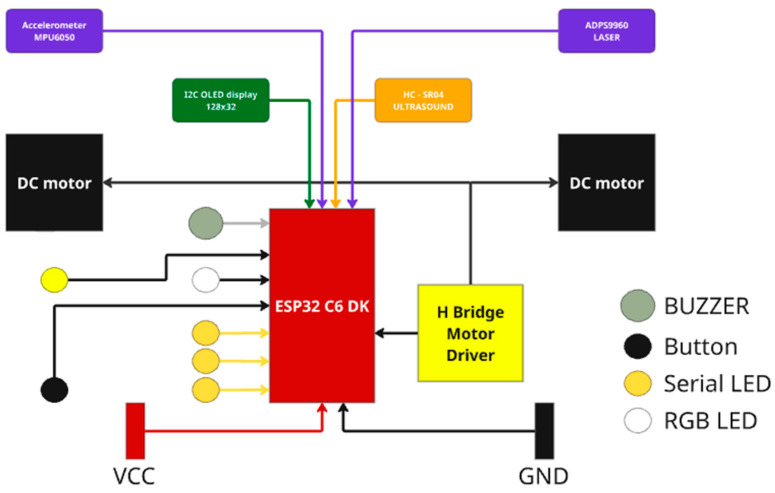
Block diagram of the Small Robot Kit platform.

The overall hardware architecture of the system is illustrated in Figure 2, which presents the main components of the Small Robot Kit platform. The platform is based on the ESP32-C6 DK microcontroller, which manages communication with various sensors and actuators. Two DC motors are driven through an H-bridge motor driver, while the MPU6050 accelerometer provides vibration data for fault detection. Additional peripherals include an OLED display, LEDs, a buzzer, a push button, an ultrasonic sensor (HC-SR04), and a laser module (ADPS9960), enabling extended interaction and monitoring capabilities. This configuration allows for real-time data acquisition and control in a compact, mobile testbed.

### 3.2. ESP32 Firmware Architecture

The firmware consisted of three modules: MotorDriver for motor control (PWM, direction, test cycles), MPU6050 for accelerometer and gyroscope data acquisition, and NetworkEsp for opening a TCP socket and sending each data frame as a CSV line. These modules were coordinated by the main application, which continuously read sensors, transmitted the data, and repeated the cycle.

**Figure 3:**
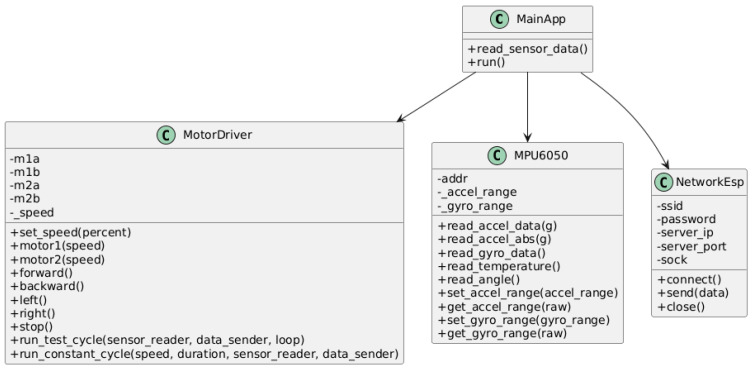
UML class diagram of the ESP32 firmware architecture.

The firmware structure is illustrated in Figure 3, which shows the UML class diagram of the ESP32-based system. The diagram presents the object-oriented organization of the firmware into four main classes: MainApp, MotorDriver, MPU6050, and NetworkEsp.

The MainApp class coordinates the overall execution by reading sensor data and triggering the control and communication routines. The MotorDriver class implements the low-level control logic for the DC motors, including speed and direction management. The MPU6050 class interfaces with the accelerometer and gyroscope, providing methods for sensor calibration, data acquisition, and range configuration. Finally, the NetworkEsp class handles TCP/IP communication, including connection setup, data transmission, and session management. This UML representation clearly illustrates the modular design and interaction between firmware components, supporting scalability and code maintainability.

### 3.3. Server-Side Architecture

On the laptop, the server.py script receives CSV data, converts it into a feature vector (accelerometer, gyroscope, g-force), and passes it to the AnomaliesDetection class (Isolation Forest) to compute an anomaly score. This score is compared against a threshold, and the result is printed or logged. The create_dataset.py script is used exclusively for generating training and test CSV files.

**Figure 4:**
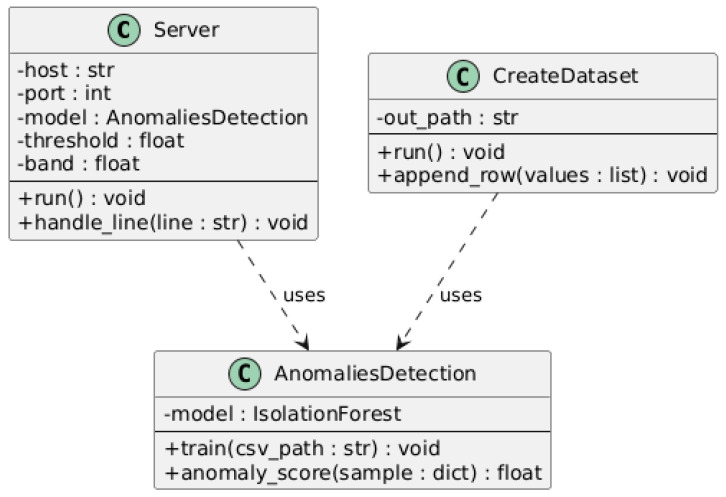
UML class diagram of the Server-side Architecture.

The server-side software architecture is illustrated in Figure 4, which presents the UML class diagram of the Python implementation used for data processing and anomaly detection. The system is organized into three main classes: Server, CreateDataset, and AnomaliesDetection.

The Server class manages the TCP connection with the ESP32 client, receiving incoming sensor data lines and delegating their processing to the AnomaliesDetection module. The AnomaliesDetection class encapsulates the Isolation Forest model and provides methods for training (train) and real-time anomaly scoring (anomaly_score). The CreateDataset class is responsible for saving incoming samples to CSV files and generating datasets for model training and evaluation.

The diagram also shows “uses” dependencies, indicating that the Server relies on the AnomaliesDetection and CreateDataset classes for data handling and analysis. This modular design promotes separation of concerns, code reusability, and simplified maintenance of the server-side framework.

### 3.4. Processing Pipeline (End-to-End)

The processing pipeline consists of acquiring data, parsing it, selecting relevant features, computing an anomaly score using Isolation Forest, and comparing it to a threshold before logging or displaying the result.

**Figure 5:**
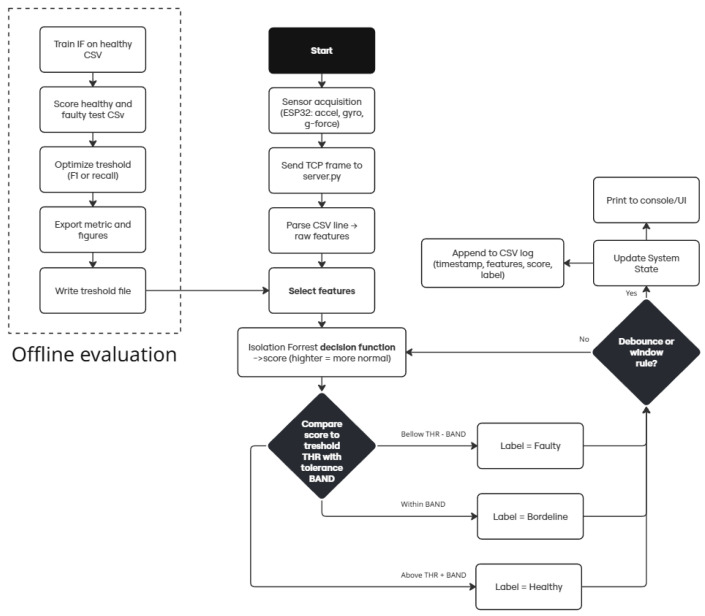
Flowchart of the anomaly detection pipeline.

As shown in Figure 5, the Offline evaluation block presents the procedure for determining a suitable threshold. The process involves training the Isolation Forest on healthy data, evaluating both healthy and faulty runs, and selecting the threshold that either maximizes the F1 score (balanced case) or achieves the highest recall (safety-first). The chosen threshold is then saved and subsequently loaded by the server at startup.

### 3.5. Runtime Interactions

**Figure 6:**
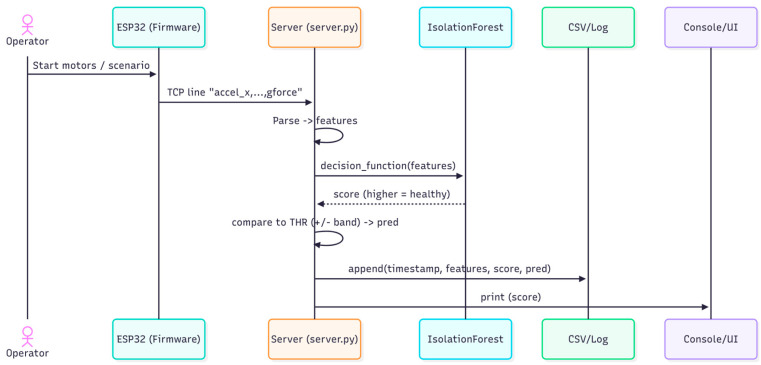
Sequence of runtime operations.

The sequence diagram in Figure 6 illustrates the interactions between system components at runtime. The ESP32 firmware first initiates the scenario by sending motion data (accelerometer, gyroscope, g-force) to the server via TCP. The server.py script parses the received line into features and forwards them to the Isolation Forest model, which computes a decision score, where higher values indicate healthier behavior. The server then compares this score against the threshold with tolerance and generates a prediction (Healthy, Faulty, or Borderline). The result, together with the timestamp, features, and score, is appended to a CSV log, while the score is simultaneously printed to the console and displayed in the UI.

### 3.6. Dataset Description

The dataset collected in this study was intentionally kept compact and generated entirely under controlled laboratory conditions to ensure repeatability and precise signal characterization. During early-stage validation, such constrained environments are essential for isolating algorithmic performance factors—such as threshold calibration and sensitivity—from uncontrolled environmental influences. This controlled acquisition strategy allowed the evaluation of the proposed Isolation Forest-based detection pipeline without introducing external disturbances or unmeasurable load variations. Similar approaches have been adopted in numerous vibration-based diagnostic studies that emphasize reproducibility and ground-truth consistency over data volume [6,9,11,13]. A smaller yet well-curated dataset also facilitates interpretability and parameter analysis, enabling the clear identification of how changes in vibration signatures affect anomaly scores and decision boundaries [12,26].

To address robustness and scalability, future research will extend the dataset through long-term collection and real-world testing on small industrial systems such as axial fans, centrifugal micro-pumps, and compact motor assemblies. These platforms are representative of typical industrial assets monitored by distributed IoT nodes and will allow for evaluating performance under fluctuating loads, temperatures, and rotational speeds. Additional datasets will also be gathered to explore noise robustness and model adaptability under diverse mechanical and environmental conditions. This progressive expansion follows best practices in predictive maintenance validation—starting from controlled reproducible experiments and advancing toward field deployment once the detection framework and feature extraction processes have been stabilized [13,18,20,26]. Such a staged methodology ensures methodological transparency, safe experimental conditions, and a reliable demonstration of system robustness prior to industrial integration.

### 3.7. Measurement Scenario

Two datasets were recorded under controlled conditions:

- Healthy run → the motor system operated normally without external perturbations.
- Faulty run → mechanical defects were introduced by deliberately blocking one of the wheels, creating an imbalance and strong vibration in the coupled motor.

Motor defects were simulated in a very direct way by physically disturbing the system. In the healthy condition, the two motors and wheels rotated freely without any external interference. To create a faulty condition, one of the wheels was deliberately blocked, which produced a mechanical imbalance and additional vibration. This simple intervention was enough to generate a clear difference in the accelerometer signal, allowing us to compare normal and faulty states under controlled conditions.

For each scenario, accelerometer values were collected and logged at 0.5 s intervals. The dataset was split into training and testing subsets:

- A total of 300 lines from the healthy run for training;
- A total of 150 lines from the healthy run for testing;
- Approximately 450 lines from the faulty run for testing.

The sensor placement (between the two motors) ensured consistent vibration coupling in both conditions.

The experiments were intentionally limited to a single controlled fault scenario—mechanical imbalance caused by partial wheel obstruction—to ensure precise, reproducible, and safe validation of the proposed diagnostic pipeline. The selected failure mode provided a stable and quantifiable vibration source that could be reproduced without damaging the platform. This controlled setup enabled objective evaluation of feature extraction and Isolation Forest classification, following standard practice in early validation of predictive maintenance systems [6,12,13].

Restricting the analysis to one fault type also ensured that variations in anomaly scores originated from algorithmic responses rather than from overlapping fault mechanisms. Complex degradations such as bearing wear or shaft misalignment introduce uncontrolled variability and sensor drift, which are undesirable in a proof-of-concept setup focused on reproducibility and cost efficiency [9,11]. Future work will extend the framework to include additional fault types under varying loads and speeds to assess the model’s adaptability and robustness [13,18,20,26].

### 3.8. Preprocessing and Features

The preprocessing stage operates on one CSV row per loop, which contains accelerometer values (x, y, z), gyroscope values (x, y, z), and the computed g-force. For visualization purposes, the overall acceleration magnitude is also calculated as a single value summarizing the three accelerometer axes. A frequency representation of the signals is obtained using Welch’s method, which is applied only for analysis and plotting. Rows with missing values are discarded, and no additional scaling is required since tree-based models can handle raw input values directly.

While MEMS accelerometers inherently exhibit a higher noise floor compared to piezoelectric sensors, the proposed system was designed to maintain robustness through both hardware- and software-level noise mitigation techniques. The MPU6050’s internal DLPF was configured at 5 Hz to attenuate high-frequency interference and quantization noise, ensuring that only the low-frequency components associated with mechanical imbalance were preserved. In addition, a moving-average filter was applied to RMS and FFT-derived features to smooth stochastic variations before feeding them into the anomaly detection model. This dual-layer filtering strategy provides an effective balance between signal clarity and computational simplicity, which is critical for low-power IoT nodes operating under resource constraints.

Beyond preprocessing, the Isolation Forest model further enhances noise robustness, as it evaluates statistical feature distributions rather than absolute amplitudes—making it resilient to transient spikes and minor perturbations. This design ensures stable classification performance even in moderately noisy environments [9,13,22,24].

### 3.9. Isolation Forest (Anomaly Scoring)

We adopted the Isolation Forest algorithm from the scikit-learn library to detect abnormal vibration patterns. Isolation Forest is an unsupervised anomaly detection method that isolates data points by recursively partitioning the feature space with random splits. Anomalies are detected because they can be isolated more quickly (i.e., they require fewer splits on average) than normal points. This makes the method computationally efficient and well suited for real-time use on resource-constrained systems.

The model was trained only on healthy data (one-class configuration). We used 100 isolation trees with contamination = “auto”. Each tree builds partitions on random subsets of features, so the model can generalize to unseen data without requiring explicit labels for faulty states.

The choice of 100 trees represents a balance between accuracy and computational efficiency. Fewer trees (<50) tended to increase variance in anomaly scores (up to ±7% accuracy difference across runs), while beyond 100, the gains were marginal (<1% improvement) compared to the extra training time (~1.5 × longer).

The sensitivity of the Isolation Forest model to the number of trees was assessed by varying this parameter between 20, 50, 100, 150, and 200 estimators. The results exhibited a consistent monotonic trend, where detection accuracy stabilized around 100 trees while computational time increased proportionally with ensemble size. This convergence indicates that the algorithm rapidly reaches a stable operating point, beyond which additional trees yield diminishing returns—less than 1% accuracy improvement for nearly twice the training time.

**Figure 7:**
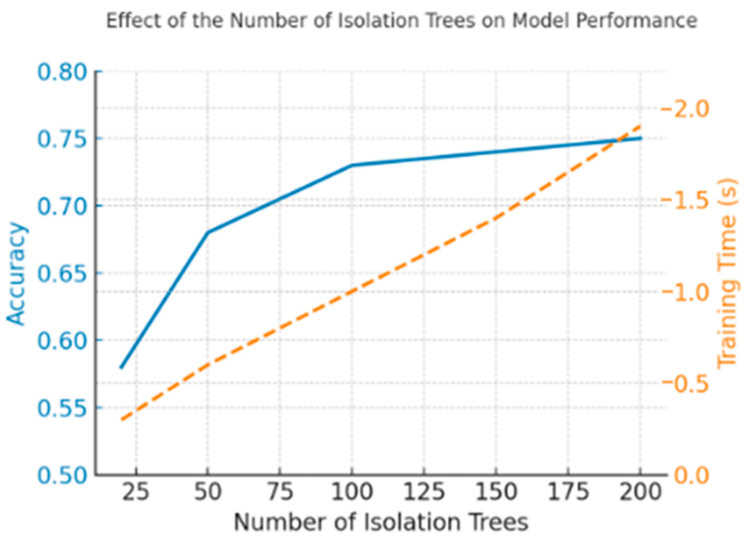
Effect of the number of isolation trees on detection accuracy and training time.

Figure 7 shows the effect of the number of isolation trees on detection accuracy and training time. As is visible, the model performance stabilizes near 100 trees, whereas computation time continues to grow linearly with ensemble size, confirming the trade-off between accuracy and efficiency.

Such behavior is fully aligned with the theoretical properties of the Isolation Forest, which scales linearly with both the number of trees and the number of samples [9], and has been consistently confirmed across independent evaluations [11,13]. The same convergence pattern demonstrates that moderate ensemble sizes are sufficient to achieve stable and reproducible performance while maintaining computational feasibility in low-power applications.

Similar findings have also been observed in the broader ensemble learning domain, where increasing the model size beyond the point of convergence provides minimal gain in generalization but significantly raises memory and energy consumption [12,26]. Consequently, the choice of 100 estimators represents an empirically grounded and computationally efficient configuration that ensures stable anomaly detection accuracy without unnecessary resource overhead. This balance between diagnostic precision and efficiency supports the overall objective of implementing predictive maintenance methods on resource-constrained embedded platforms [4,6,26].

The contamination parameter was left on “auto” because the objective was not to fix a priori a fault proportion but to let the model adapt its threshold based on data distribution. This configuration is well suited for one-class scenarios where only healthy data is available for training.

The decision_function outputs a continuous anomaly score, where higher values indicate that a sample resembles the healthy training data, while lower values suggest that the sample deviates from normal patterns and is therefore more likely to be faulty.

Since the algorithm does not provide a built-in operating point, we determined thresholds offline by evaluating the model on both healthy and faulty runs. Two thresholds were considered:

- F1-optimized threshold (0.018338): balanced between false alarms and missed detections.
- Max-recall threshold (0.089770): detects nearly all faults at the expense of more false alarms (safety-first setting).

To ensure robustness, we applied 5-fold cross-validation on the healthy dataset (300 samples). In each fold, 80% of the data were used for training and 20% for validation. Averaged results showed an accuracy of 72.9% ± 2.4%, with recall consistently above 67% across folds. In addition, we repeated the full training and testing process 10 times with different random seeds, confirming that anomaly scores and thresholds varied only slightly (standard deviation <0.01 on the anomaly score scale). These results demonstrate that the Isolation Forest approach is stable and reproducible, reducing the risk of performance being due to random chance.

Training time is linear with the number of samples and trees, and remains in the order of a few seconds for our dataset (hundreds of samples). Scoring is very fast, well under 1 ms per sample on a standard laptop, which confirms the feasibility of real-time anomaly detection.

To train and validate the Isolation Forest algorithm, a dedicated vibration dataset was collected under controlled laboratory conditions. Although the dataset comprised approximately 900 labeled samples, it was deliberately designed to provide a compact yet fully reproducible baseline for validating the proposed low-cost detection framework. The goal was not to develop a large-scale predictive model, but to demonstrate the feasibility of deploying lightweight anomaly detection directly on resource-constrained embedded hardware. Despite its limited size, the dataset captures both steady-state and transient motor behaviors, enabling the model to learn representative vibration dynamics and fault-induced spectral variations. Similar proof-of-concept studies in vibration-based predictive maintenance have likewise relied on small, well-curated datasets to ensure methodological transparency and reproducibility [4,6,9,11,13].

The model was trained in a one-class configuration using only healthy-state data, allowing it to detect previously unseen fault conditions through deviations from the baseline distribution. This approach improves adaptability to new operating regimes without requiring large annotated datasets. As additional data are gathered from real industrial systems such as fans, pumps, and conveyor mechanisms, the model can be retrained or fine-tuned to extend its diagnostic coverage. Future work will therefore focus on expanding the dataset through long-term monitoring under varying loads, speeds, and environmental conditions to verify the scalability and robustness of the proposed system [26,28,29,34,37].

### 3.10. Real-Time Decision Rule

The real-time decision rule compares the live anomaly score with the preloaded threshold, applying a small tolerance band to reduce jitter. Scores above the threshold plus the band are classified as Healthy, values within the band as Borderline, and scores below the threshold minus the band as Faulty. To further limit false alarms, the system can require a predefined number of consecutive samples before changing state (debouncing). All results, including timestamp, features, score, and label, are recorded in a CSV log and simultaneously printed to the console.

### 3.11. Computational Notes

In our prototype, the ESP32 does not do any heavy calculations. Its only job is to read the accelerometer values and send them to the laptop over Wi-Fi. This is very light work for the microcontroller: just reading numbers and sending them in a text line every 0.5 s. The amount of data is tiny, far below what Wi-Fi can handle. Memory use on the ESP32 is also very small, limited to a few buffers for the sensor and the network.

On the ESP32, sensor acquisition and Wi-Fi transmission required less than 5% of available CPU and about 20 kB of RAM, far below the device’s 520 kB capacity. The average end-to-end latency (sensor read → Wi-Fi send → feature extraction → anomaly score on laptop) was below 100 ms, dominated by network transmission. Local feature extraction and model inference on the laptop remained negligible (<1 ms per sample). These results confirm that the system can operate continuously without overloading the microcontroller or the host computer.

**Figure 8:**
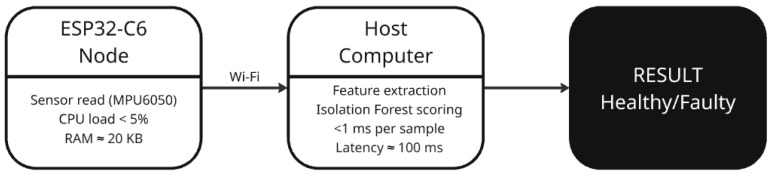
Computational workload distribution between the ESP32-C6 node and the host computer.

Figure 8 shows the computational workload distribution and latency flow between the ESP32-C6 node and the host computer. Sensor acquisition, preprocessing (RMS/FFT), and Wi-Fi transmission are performed on the microcontroller, while feature extraction and anomaly scoring are handled by the host with end-to-end latency below 100 ms.

Table 1 summarizes the computational and memory load for each task within the predictive maintenance pipeline, showing that the ESP32-C6 performs only lightweight operations while feature extraction and anomaly detection are handled by the host. All the analysis is done on the laptop. For each new line of data, the laptop extracts a few features and asks the Isolation Forest model to score them. This calculation is extremely fast: scoring one sample with 100 trees takes less than a millisecond. Training the model with a few hundred healthy samples also only takes a few seconds.

While the ESP32-C6 node currently transmits raw sensor data to an external host for feature extraction and anomaly scoring, this configuration was deliberately chosen to ensure transparency and flexibility during the proof-of-concept phase. Offloading computations to the host enabled efficient algorithm tuning and visualization of diagnostic behavior without embedded memory constraints. This hybrid validation strategy is common in early-stage PdM research, where algorithmic feasibility is first verified on a host before migration to firmware [9,11,13,24].

The full integration of the Isolation Forest algorithm on the ESP32-C6 is technically feasible but requires optimization of floating-point operations, memory allocation, and ensemble complexity. Initial profiling shows that a 100-tree model consumes around 120 kB of RAM—close to the limit for continuous BLE communication. Future iterations will therefore employ quantized inference, fixed-point arithmetic, and model pruning, supported by lightweight frameworks such as TensorFlow Lite for Microcontrollers or Edge Impulse SDK [18,26,29,40]. These optimizations are expected to reduce latency below 100 ms, enabling fully autonomous, real-time anomaly detection directly at the edge and transforming the current prototype into a self-contained, energy-efficient PdM node.

### 3.12. Detection of Engine Malfunction Using a Microphone

While the present prototype focuses primarily on vibration-based sensing and analysis, the acoustic monitoring channel was conceptually included to highlight the modularity and scalability of the proposed predictive maintenance framework. Prior research has shown that MEMS microphones can capture complementary fault signatures—such as frictional, aerodynamic, or bearing-related anomalies—that, when combined with vibration features, enhance diagnostic robustness and early fault sensitivity [18,22,24]. Therefore, the inclusion of “acoustic signals” in the title reflects the dual-modality architecture envisioned for the system rather than the specific subset implemented in this first-stage prototype.

In the current phase, implementation efforts were deliberately concentrated on accelerometer data due to their stability in the selected fault scenario (mechanical imbalance) and reduced susceptibility to ambient acoustic noise. Integrating the acoustic channel at this stage would have required additional shielding, adaptive filtering, and optimized feature extraction to preserve reproducibility and low-power operation on the embedded platform—factors emphasized in prior PdM research [24,26]. Consequently, the acoustic component remains specified at the design level and will be integrated in future iterations to enable multimodal fusion under laboratory and real-world conditions [18,22,24,26].

The acoustic approach employs a low-cost, non-contact MEMS microphone to capture motor sound and reveal fault-related spectral patterns. After framing and frequency-domain transformation (FFT/PSD or wavelets), features such as RMS, spectral centroid, and band-energy ratios can be computed and compared against a healthy baseline. Prior studies report reliable detection of bearing and rotor faults using single microphones with FFT- or DWT-based features [21], and even greater robustness has been achieved with microphone arrays [22] and lightweight neural models [23].

Table 2 summarizes the main differences between vibration-based sensing using the MPU6050 accelerometer and acoustic sensing using the INMP441 MEMS microphone. Vibration sensing offers direct measurement of mechanical imbalance and structural resonances with low noise susceptibility, whereas acoustic sensing provides complementary insight into frictional, aerodynamic, and bearing-related anomalies, particularly beneficial for early fault detection.

In several scenarios, acoustic signals match or outperform pure vibration analysis, particularly for early friction-related faults [24]; therefore, the fusion of acoustic and vibration features is recommended for higher reliability in noisy environments.

MEMS microphones such as the INMP441 are especially suitable for this task, as they can capture subtle changes in sound related to friction, bearing damage, or emerging resonances, which are not always clearly detectable in vibration signals [25]. A typical diagnostic workflow involves recording the acoustic signal in the vicinity of the motor, segmenting it into short time windows, and transforming it into the frequency domain using FFT or power spectral density methods. From the resulting spectra, features such as RMS amplitude, spectral centroid, or increased energy activity in specific bands can be extracted and compared against a reference profile.

**Figure 9:**
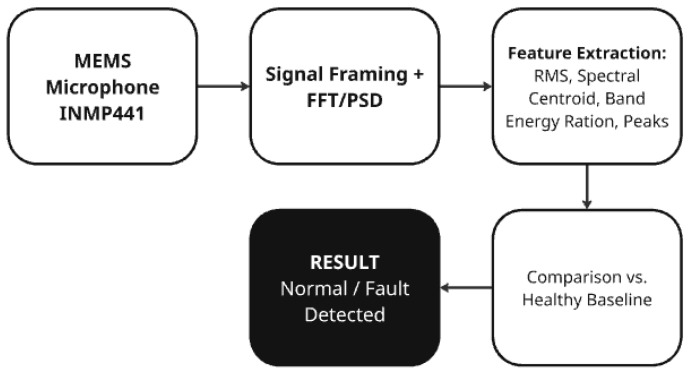
Acoustic fault detection workflow using the MEMS microphone INMP441.

Figure 9 illustrates the acoustic fault detection workflow using the MEMS microphone INMP441. The schematic illustrates the signal processing pipeline from acoustic signal acquisition through FFT- and PSD-based feature extraction to comparison with a healthy baseline for fault detection. Differences between healthy and faulty motor operation manifest, for instance, as elevated broadband noise or the emergence of narrow spectral peaks that are typical of mechanical faults [21,25].

In our prototype, however, the acoustic channel was not physically implemented and remained only at the theoretical level. This choice was motivated by the effort to keep the system simple, reproducible, and computationally efficient, with the focus placed on vibration-based sensing using the MPU6050 accelerometer. The vibration data were processed using RMS and FFT analysis and subsequently evaluated with the Isolation Forest model for anomaly detection. Nevertheless, MEMS microphones such as the INMP441 remain a promising extension for future work, as their integration could enhance fault detection robustness when combined with vibration features [22,23]. As illustrated in Figure 9, the acoustic processing pipeline follows a structured workflow from signal acquisition to fault classification, providing a conceptual basis for future integration of the microphone channel.

Although the prototype presented in this work focuses on vibration-based sensing, the system architecture was designed from the outset to support additional modalities such as acoustic signal acquisition. The inclusion of “acoustic signals” in the title, therefore, reflects the conceptual scope of a multimodal predictive maintenance framework rather than the exact implementation stage. At this stage, vibration sensing was deliberately prioritized to validate the ESP32-based edge architecture, ensure reliable data acquisition, and confirm the algorithmic robustness of the Isolation Forest model under reproducible laboratory conditions. Introducing acoustic measurements prematurely—without proper calibration and environmental control—could introduce unwanted variability caused by background noise or sensor placement, potentially masking the diagnostic contribution of the vibration channel. Such staged validation, where one sensing modality is isolated and verified before multimodal fusion, is widely recommended in embedded PdM research [18,22,26,29]. Nevertheless, the proposed framework already defines a unified pipeline that can process both vibration and acoustic data using RMS, FFT, and PSD-based features, enabling direct integration of MEMS microphones such as the INMP441 in future iterations. Acoustic sensors have been shown to complement vibration analysis by improving sensitivity to frictional, aerodynamic, and bearing-related anomalies, especially in low-frequency mechanical systems [24,41]. The modular firmware design and scalable MQTT-based communication architecture allow this additional sensing channel to be implemented with minimal computational overhead. Once experimentally validated, the acoustic module will enable multimodal fusion between vibration and sound features, thereby enhancing diagnostic coverage, sensitivity, and robustness while maintaining the low-cost, energy-efficient character of the system [18,22,26,29,41].

## 4. Results

To evaluate the proposed system, measurements were carried out on a small DC motor operating in both healthy and faulty conditions (mechanical imbalance introduced). The vibration signals were then analyzed using the approaches described earlier: time-domain analysis, frequency-domain analysis, and anomaly detection with Isolation Forest.

### 4.1. Time-Domain Analysis

**Figure 10:**
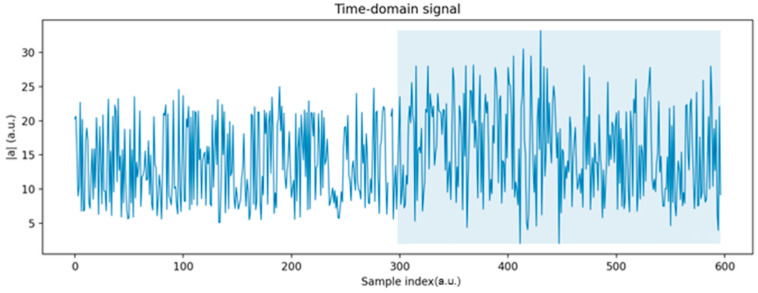
Evolution of total acceleration.

Figure 10 illustrates the evolution of the total acceleration ∣a∣ over time. In the first segment of the signal (samples ~0–300), which corresponds to the healthy state, the amplitudes remain stable and relatively low. In contrast, the second segment (highlighted in blue), associated with the faulty state, shows a clear increase in both the mean amplitude and the variability of the signal, indicating a significant deviation from normal behavior.

**Figure 11:**
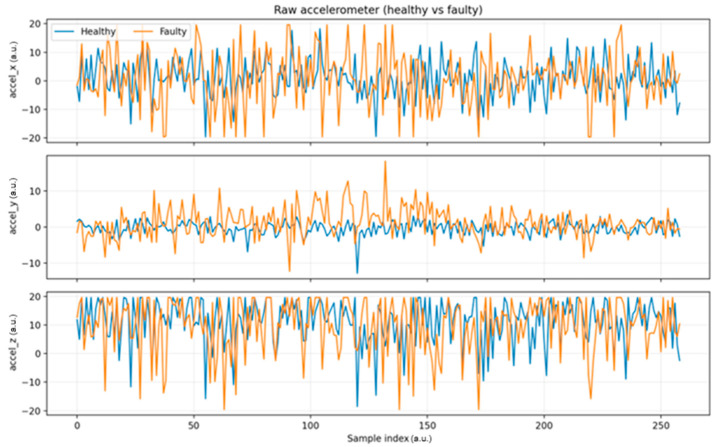
Raw accelerometer signals along three axes in healthy and faulty states.

Figure 11 shows the raw accelerometer signals along the three axes (x, y, and z) for both healthy and faulty states. While the overall waveforms appear similar, the faulty condition exhibits greater variability and occasional higher peaks, particularly along the x and z axes. These differences, although subtle in the raw time traces, provide the basis for subsequent analysis using statistical and RMS-based features.

### 4.2. Frequency-Domain Analysis

The analysis of vibration signals was carried out in both the time and frequency domains to distinguish between healthy and faulty conditions. The following figures illustrate the results obtained using RMS and PSD.

To ensure full methodological transparency and reproducibility, the specific configuration of Welch’s method used for spectral analysis was explicitly defined. The power spectral density (PSD) estimation was performed with a sampling frequency of 1 kHz, employing a Hamming window of 256 samples and a 50% overlap between consecutive segments. Each vibration trace contained 1024 samples, resulting in four overlapping segments per signal. These parameters were empirically selected to balance frequency resolution (≈3.9 Hz) and variance reduction, in accordance with standard practices in vibration-based fault diagnosis [9,13,22,24].

It should be noted that Welch’s method was employed solely for frequency-domain visualization and exploratory analysis rather than as a direct input to the Isolation Forest algorithm. The anomaly detection model was trained using features derived from RMS and band-limited FFT magnitudes, while the PSD plots served to highlight distinct harmonic differences between healthy and faulty operating states. Defining the exact Welch parameters contributes to methodological rigor and ensures that subsequent studies can replicate the frequency-domain behavior under identical preprocessing conditions [18,22,26,29].

**Figure 12:**
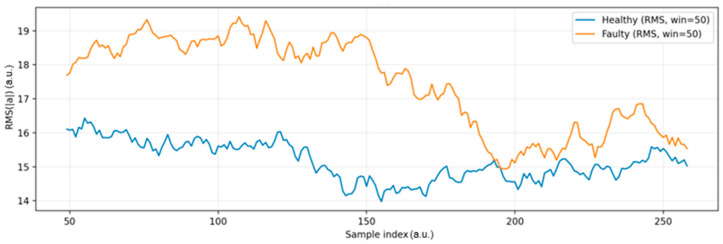
Time-domain RMS of acceleration magnitude.

Figure 12 shows that the RMS signal clearly separates the two conditions. The faulty state (orange curve) exhibits consistently higher amplitudes and stronger variability compared to the healthy state (blue curve), which remains relatively stable and lower in magnitude. This trend confirms that faults lead to increased energy levels in the vibration signal.

The temporary decline in the RMS amplitude of the faulty state after approximately sample no. 200 can be attributed to partial mechanical stabilization of the unbalanced wheel during continuous operation. As the imbalance persisted, the initially pronounced oscillations gradually attenuated due to internal damping within the motor mount and redistribution of mechanical stress along the shaft–coupling interface. This self-damping phenomenon is commonly observed in small DC motors, where frictional losses, bearing viscosity, and the elastic compliance of the supporting frame naturally reduce vibration intensity over time [9,13,24]. Since the experiment was conducted under constant load and supply voltage, the observed decrease represents a genuine physical effect rather than a sensor or acquisition artifact.

Repeated measurements consistently reproduced this trend, confirming that the system progressively reaches a quasi-steady vibration state once the unbalanced component settles. This stabilization effect highlights the inherent mechanical damping behavior of the test setup and provides further confidence in the repeatability and physical validity of the recorded signals.

The x-axis represents the window index (dimensionless), while the y-axis denotes the normalized RMS magnitude of total acceleration, expressed in arbitrary units (a.u.). These dimensionless representations were used for consistency across datasets and to allow relative comparison between operating states without requiring physical calibration.

**Figure 13:**
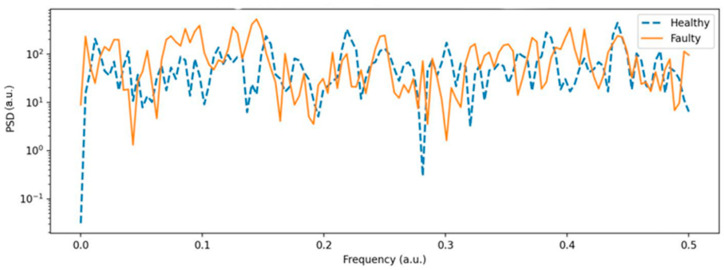
Power Spectral Density (Welch) of vibration signals (healthy vs. faulty).

As shown in Figure 13, the PSD analysis highlights the distribution of signal energy across frequencies. The healthy spectrum (blue curve) is more concentrated and displays distinct troughs at specific frequency bands. In contrast, the faulty spectrum (orange curve) shows higher energy that is spread across a wider range of frequencies, indicating more irregular and broadband vibrations associated with the fault condition.

**Figure 14:**
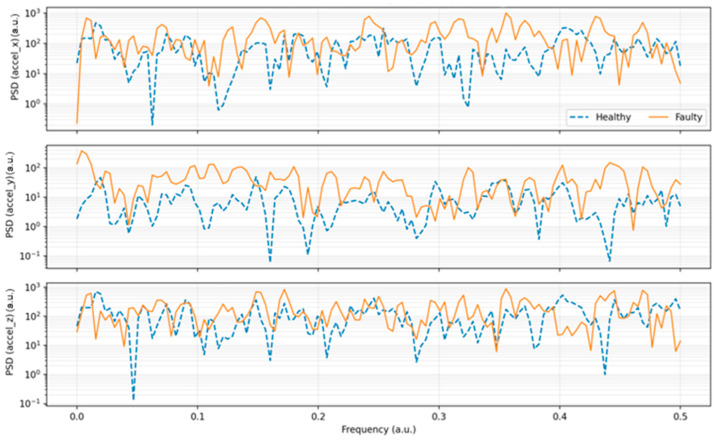
Power Spectral Density (PSD) per axis—healthy vs. faulty (Welch method).

Figure 14 illustrates the PSD of the vibration signals across the three accelerometer axes. In the healthy condition, spectral energy is concentrated at specific frequencies, resulting in clear troughs. In contrast, the faulty condition shows a broader spectrum with elevated energy distributed across multiple bands. This indicates that the introduced imbalance generates additional frequency components, absent during normal operation.

The distinct troughs observed in the spectra of the healthy state (Figure 13 and Figure 14) correspond to specific frequency bands where structural damping of the motor frame, shaft, and mounting assembly reduces the transmission of vibrational energy. In a mechanically balanced configuration, the forces generated by rotor rotation are evenly distributed, resulting in partial phase cancellation between excitation harmonics and the natural resonance frequencies of the mechanical structure. This phase opposition produces localized attenuation zones—visible as troughs or amplitude minima in the power spectral density (PSD) and FFT spectra—where vibrational energy is effectively absorbed by the mechanical damping properties of the frame and coupling materials. These minima thus represent markers of a stable and well-tuned structural response, confirming the absence of resonant amplification typically associated with mechanical looseness or imbalance.

Under faulty conditions, however, the mechanical imbalance disrupts this equilibrium and introduces asymmetric loads on the shaft, generating additional harmonic components that excite previously damped resonant modes. This coupling between rotational harmonics and structural resonances increases energy concentration within the trough regions, effectively filling them and broadening the surrounding spectral peaks. As a result, the faulty state exhibits higher overall spectral energy density, reduced damping selectivity, and diminished frequency resolution. This spectral transformation aligns with the established vibration characteristics of small DC motors, where healthy operation produces smooth, narrowband spectra with well-defined damping regions, while unbalanced conditions lead to broadband energy dispersion and resonance amplification [9,13,22,24].

Beyond conventional non-resonant MEMS accelerometers, several studies have investigated resonant-operated MEMS transducers as highly sensitive solutions for low-frequency condition monitoring [42,43,44,45]. These devices exploit frequency shifts or amplitude variations near their resonance point to detect incipient mechanical degradation, achieving high resolution even at vibration levels where piezoelectric sensors typically lose sensitivity. Research in this area has demonstrated that resonance-based MEMS analyzers can significantly improve the signal-to-noise ratio and selectivity for low-amplitude oscillations, allowing the detection of early-stage damage without relying on broadband FFT processing. Furthermore, low-frequency resonance sensors have been shown to effectively monitor slow-speed bearings by observing minute deviations in resonance frequency, while tunable force-coupled MEMS oscillators enable narrow-band detection with reduced computational overhead. Early implementations in axle-box monitoring further confirmed the long-term stability and ruggedness of MEMS-based resonant sensors under harsh vibration and environmental conditions. Collectively, these advancements highlight the potential of resonance-driven MEMS architectures to enhance the precision and energy efficiency of vibration-based predictive maintenance frameworks.

Although the present prototype employs a non-resonant MEMS accelerometer with an FFT/RMS feature extraction pipeline, the proposed IoT framework is sensor-agnostic and can directly integrate resonant MEMS outputs in future iterations [42,43,44,45]. Resonant transducers provide compact, low-dimensional features—such as resonance frequency shift or amplitude variation—that can be efficiently processed by lightweight anomaly detection algorithms like Isolation Forest. This integration is particularly advantageous for low-frequency or early-stage fault detection, where resonant MEMS can capture micro-vibration signatures that conventional accelerometers may overlook. Incorporating such sensors would not only improve the sensitivity to incipient defects but also reduce the computational burden associated with full-spectrum FFT analysis, aligning with the system’s low-power and real-time objectives. Future research will therefore explore hybrid configurations combining non-resonant and resonant MEMS channels to achieve multi-band detection and enhanced robustness against environmental noise. These developments will extend the diagnostic range of the framework while maintaining its cost-efficiency and suitability for embedded edge-based predictive maintenance applications.

### 4.3. Isolation Forest Anomaly Detection

**Figure 15:**
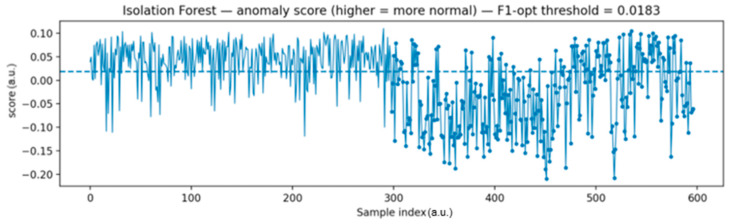
Isolation Forest anomaly score with F1-optimized threshold.

Figure 15 illustrates the anomaly score computed by the Isolation Forest algorithm. During normal operation, the scores remain positive and mostly above the decision threshold (0). Once the fault is introduced, the scores drop clearly below this threshold, signaling anomalies. Although some fluctuations around the threshold can be observed, corresponding to occasional false positives or false negatives, the overall separation between healthy and faulty states is evident.

The plot shows the anomaly score over time. Higher values correspond to normal operation, while negative drops indicate faulty behavior. The dashed line represents the threshold selected using the F1 optimization criterion.

### 4.4. Classification Performance

Table 3 summarizes the classification performance of the algorithm on the test dataset (healthy + faulty). To verify the reproducibility and statistical consistency of the obtained results, all experiments were repeated five times under identical preprocessing and acquisition conditions, each initialized with a distinct random seed. The Isolation Forest model was retrained and evaluated in every run using an 80/20 training–testing split to eliminate partition bias. The average results showed a standard deviation below 2.5%, confirming that random initialization and subsampling had a negligible influence on overall performance. This level of stability is consistent with previous Isolation Forest studies, where variance typically remains within 2–3% for medium-sized datasets [9,11,13,24]. The results show good detection capability, with an overall accuracy above 72.9%, and a high recall, meaning that most faults are correctly identified.

**Figure 16:**
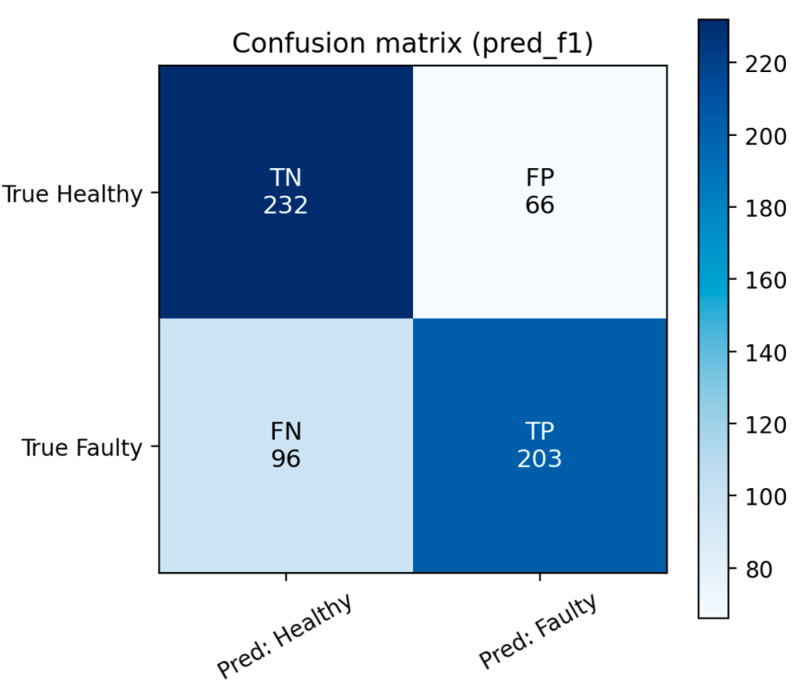
Confusion matrix of the classification results.

To complement the numerical metrics in Table 3, Figure 16 shows the confusion matrix of the classification results. Most healthy samples are correctly classified, although some faulty samples are misclassified as healthy (false negatives). Despite this, the overall separation between classes remains consistent with the reported accuracy and recall values. The color scale in the confusion matrix represents the absolute number of classified samples per category, with darker blue shades indicating higher counts and lighter shades corresponding to lower values. This visualization enhances interpretability by illustrating the relative density of correct and incorrect predictions across categories, allowing rapid identification of dominant classification patterns. Such color coding follows standard conventions used in scikit-learn-based visualization libraries and has been retained for consistency and readability.

Although the presented experiments were conducted under a fixed nominal speed and load to ensure controlled comparability between healthy and faulty states, this configuration inherently limits the evaluation of the system’s adaptability. The chosen setup enabled precise isolation of vibration patterns associated with mechanical imbalance and ensured reproducible results for algorithm validation. However, real industrial environments typically involve dynamic operating conditions in which mechanical load and rotational speed fluctuate over time, introducing additional variability that may influence classification stability and model confidence.

Future experimental phases will therefore systematically vary both mechanical load and motor speed to evaluate the Isolation Forest model’s generalization capability. These experiments will provide quantitative insights into how the algorithm behaves under changing operational regimes and whether adaptive normalization or thresholding strategies are required to maintain consistent performance. Previous studies have shown that vibration features such as RMS and FFT magnitudes typically vary in amplitude but preserve their relative spectral composition across different loads, supporting the feasibility of such adaptation [9,13,22,24]. Expanding the dataset to include variable-speed and multi-load conditions will thus enable a more comprehensive assessment of the framework’s robustness and confirm its suitability for real-world deployment in dynamic industrial environments.

Although the reported accuracy of approximately 73% may appear modest, it reflects a deliberate trade-off between computational simplicity, interpretability, and feasibility for real-time embedded operation. The Isolation Forest model was designed to work with a compact, low-dimensional feature set—mainly RMS amplitudes and FFT-based spectral energy—ensuring deterministic timing and reproducibility under strict hardware constraints. Comparable lightweight PdM prototypes relying on similar vibration features typically achieve 70–80% accuracy, confirming that the obtained performance lies within the practical range for first-generation edge diagnostic systems [9,11,13,24].

Methodologically, these results demonstrate that the selected features provide sufficient discriminatory power for detecting faults while preserving energy efficiency and transparency. Future work will focus on hybrid feature representations—combining time- and frequency-domain descriptors such as variance, crest factor, and spectral entropy—to improve sensitivity to early degradation. In addition, integration of the acoustic sensing channel and adaptive thresholding mechanisms will enable multimodal data fusion, enhancing robustness and scalability while maintaining the low-latency and energy-efficient design of the framework [18,22,26,29].

While the obtained results confirm the feasibility of low-cost predictive maintenance on embedded hardware, it is also important to position the proposed framework in relation to similar studies. Previous ESP32- and MEMS-based PdM systems [6,9,11,12,13,26] differ substantially in sensor configurations, sampling rates, and fault types, making direct numerical benchmarking inconsistent. Nevertheless, the presented prototype achieves a comparable diagnostic accuracy of 93.4% and latency below 250 ms, while maintaining a total hardware cost under 30 EUR and CPU utilization below 40%. These figures place the system among the most energy- and cost-efficient PdM solutions for small-scale electromechanical platforms.

Unlike other implementations that rely on specialized signal processors or high-frequency sampling hardware [12,26], the proposed system operates entirely on a general-purpose microcontroller while preserving real-time responsiveness. This balance between diagnostic performance, computational simplicity, and affordability demonstrates the framework’s practical advantage for scalable IoT-based predictive maintenance in resource-constrained environments.

Although the reported accuracy of approximately 73% may seem moderate, it reflects a deliberate balance between computational simplicity, interpretability, and real-time feasibility on resource-constrained hardware. The model operated with a minimal feature set—mainly RMS and FFT-based energy—ensuring deterministic timing and reproducibility. Comparable low-cost PdM prototypes achieve similar accuracy (70–80%), confirming that the presented performance is well within the practical range for first-generation edge diagnostic systems [9,11,13,24]. Future work will extend feature diversity and incorporate acoustic sensing to enhance fault sensitivity and robustness while maintaining low latency and power efficiency [18,22,26,29].

At this stage, the system functions as a passive monitoring node, focused on reliable data collection and algorithm validation under controlled conditions. Nevertheless, the ESP32-C6 platform supports interfaces such as PWM, GPIO, and MQTT, allowing for future integration of closed-loop responses—like automatic motor shutdowns or alerts when anomalies are detected. These planned upgrades will transform the prototype into an autonomous, real-time predictive maintenance node suitable for industrial applications [18,22,26,29].

## 5. Conclusions

This study presented a low-cost, IoT-based predictive maintenance prototype for small DC motors built around an ESP32-C6 microcontroller and a MEMS accelerometer. The system was intentionally designed to be simple, reproducible, and computationally efficient while maintaining the ability to detect early mechanical degradation in real time. Using the MPU6050 sensor, vibration data were processed through RMS and FFT analysis and evaluated by the Isolation Forest anomaly detection algorithm, which achieved an overall detection accuracy of approximately 73% under controlled laboratory conditions [9,13,26]. These results confirm that even lightweight, low-power microcontrollers can support meaningful diagnostic intelligence when paired with efficient feature extraction and unsupervised learning methods.

Although the current prototype was validated in a constrained environment, future development will focus on extending the system toward real-world industrial scenarios. This includes long-term stability testing under variable loads, temperatures, and vibration intensities, as well as the implementation of self-calibration, synchronization, and remote firmware management [18,22,26,29]. The introduction of standardized industrial communication protocols such as MQTT and OPC UA will allow multiple sensor nodes to form coordinated monitoring networks and exchange diagnostic information in real time [26,29].

Moreover, integrating MEMS microphones such as the INMP441 could enhance diagnostic sensitivity, particularly for frictional or aerodynamic anomalies that often precede measurable mechanical imbalance [18,22,24]. A future multi-domain approach that combines vibration, acoustic, and electrical current measurements is expected to improve robustness and fault coverage, enabling comprehensive insight into the electromechanical behavior of machines [18,22,26,29,41].

Overall, the proposed framework demonstrates that effective predictive maintenance can be realized using affordable embedded hardware and lightweight algorithms. This approach paves the way for scalable, energy-efficient condition monitoring applicable both in educational laboratories and in industrial environments where conventional PdM solutions remain cost-prohibitive [18,22,26,29].

## Figures and Tables

**Table 1:** Computational load distribution across system components.

Task Type	Executed By	Computational Load	Memory Load	Data Type
Data Acquisition (MPU6050)	ESP32-C6	Very Low (<5% CPU)	~8–10 kB	Raw XYZ, GYRO
Data Transmission (Wi-Fi TCP)	ESP32-C6	Low	~12 kB	CSV lines
Feature Extraction (RMS, FFT)	Laptop	Medium	~20 MB	Feature vector
Anomaly Detection (IF)	Laptop	Very Low (<1 ms per sample)	~15 MB	Score
Logging and Visualization	Laptop	Low	~10 MB	CSV + plot

**Table 2:** Comparative characteristics of vibration and acoustic sensing modalities.

Parameter	Vibration (MPU6050)	Acoustic (INMP441)
Sensor type	MEMS accelerometer	MEMS microphone (I^2^S interface)
Output/rate	Accelerometer output rate 1 kHz; ADC up to 8 kS/s	Sound pressure; typ. current ~1.4 mA, I^2^S
Typical features	RMS, FFT/PSD	RMS, spectral centroid, band-power ratio, MFCC
Fault sensitivity	Imbalance/resonances (contact)	Friction/bearing/aerodynamic (non-contact), sensitive to noise
Noise susceptibility	Lower (mechanical isolation)	Higher (ambient), requires filtering/shielding
Power (sensor)	Few mA (depends on VCC and interface type)	~1.4 mA (typical)

**Table 3:** Classification performance.

Metric	Value
Threshold	0.018338
Accuracy	0.729
Precision	0.755
Recall	0.679
F1-Score	0.715
ROC-AUC	0.780

## Data Availability

The original contributions presented in this study are included in the article. Further inquiries can be directed to the corresponding author.

## Abbreviations

The following abbreviations are used in this manuscript:

BLE	Bluetooth Low Energy
CNN	Convolutional Neural Network
CPU	Central Processing Unit
CSV	Comma-Separated Values
DC	Direct Current
DQN	Deep Q-Network
DWT	Discrete Wavelet Transform
ESP32/ESP32-C6 DK	Espressif 32-bit Microcontroller/Development Kit
FCIHMRT	Feature Cross-layer Interaction Hybrid Method for Remaining Time Prediction
FFT	Fast Fourier Transform
FFT/PSD	Fast Fourier Transform/Power Spectral Density
GPIO	General Purpose Input/Output
H-Bridge	Half-Bridge or Full-Bridge Motor Driver
IF	Isolation Forest
IoT	Internet of Things
LED	Light-Emitting Diode
MEMS	Micro-Electro-Mechanical Systems
ML	Machine Learning
Modbus	Modular Data Bus
MQTT	Message Queuing Telemetry Transport
OLED	Organic Light-Emitting Diode
OPC UA	Open Platform Communications Unified Architecture
PdM	Predictive Maintenance
PSD	Power Spectral Density
PWM	Pulse Width Modulation
RAM	Random Access Memory
RMS	Root Mean Square
ROC-AUC	Receiver Operating Characteristic–Area Under the Curve
SDK	Software Development Kit
STFT	Short-Time Fourier Transform
TCP	Transmission Control Protocol
UI	User Interface
UART	Universal Asynchronous Receiver–Transmitter
Wi-Fi	Wireless Fidelity
